# Effects of Digital Versatile Disc (DVD)-Based Home Exercise Programs on the Physical Function of Community-Dwelling Older Adults: A Pilot Study

**DOI:** 10.7759/cureus.98082

**Published:** 2025-11-29

**Authors:** Takashi Maeda, Minori Katada, Tomoko Uchida, Atsuko Hayashi

**Affiliations:** 1 Graduate School of Health Sciences, Kobe University, Kobe, JPN

**Keywords:** dual task, exercise, functional performance, older adults, single task

## Abstract

Objective

This study aimed to examine the effects of a digital versatile disc (DVD)-based home exercise program comprising either a physical exercise-only task (single-task (ST)) or a cognitive and physical exercise task (dual-task: DT) on the physical functions of community-dwelling individuals. There are few reports on DVD-based ST and DT training for improving physical function at home. Further, we investigated the effects of ST and DT exercises on sustaining and improving physical function.

Methodology

Thirty-three participants were included in this study. Seventeen and 16 participants were allocated to the ST and DT training groups, respectively. The participants were instructed to engage as much as possible with each DVD exercise program at home for four months. To assess physical function, left and right grip strength, the timed-up-and-go (TUG) test, and the two-step test (Locomo) for locomotive syndrome were measured before and after the intervention.

Results

The two groups showed no significant differences in grip strength, TUG, or Locomo scores before and after the intervention. In the comparison between before and after the intervention within the groups, there was a significant difference in the TUG test results for the ST group (*P* = 0.03). However, there were no significant differences in the other measurements. In the DT group, no items showed significant differences before and after intervention.

Conclusions

The results suggest that an ST of physical exercise effectively improves physical function while engaging in DVD exercises at home and that a dual task may also sustain physical function.

## Introduction

It has been reported that aging causes declines in muscle strength, balance, walking ability, and cognitive function. Age-related decline in physical and cognitive functions in older adults increases the risk of falls and dependency on activities of daily living [1]. Therefore, it is important to prevent physical and cognitive decline associated with aging.

Regular exercise appears to be effective in sustaining and improving physical and cognitive functions. Group-based exercise programs are recommended as health-promoting methods for community-dwelling older adults [2-4]. Previous studies have reported that a multifactorial exercise program that combines both aerobic and balance training is particularly important. The importance of intervention programs involving both physical exercise and cognitive tasks has also been highlighted. Programs that combine cognitive tasks with physical exercise are known as dual-task (DT) training [5,6]. In a study targeting people over 60 years old, DT training has partially improved cognitive functions such as immediate recall [7] and effectively prevented falls [8]. Moreover, Li et al. examined the effects of DT intervention on physical function and concluded that DT training could improve standing balance even if the training comprised only cognitive DTs [9].

Although several effective intervention methods for the physical, mental, and cognitive aspects of older adults have been investigated, most of these studies are limited to group exercises. In many cases, these studies were conducted in institutional facilities with equipment, and the participants often had no difficulty walking in their community. In some cases, the instructors tailored individual exercises for each participant. When targeting older adults with difficulty in walking or accessing a facility, the exercise itself may be challenging. The COVID-19 pandemic has prevented people from leaving and gathering in groups. To maintain a healthy physical state under such circumstances, it is necessary to consider how exercise programs can be provided, assuming cases in which it is impossible to tailor individualized exercises in face-to-face situations. Furthermore, developing and practicing new exercise programs without standing or walking would be necessary and effective, particularly for people who require assistance with walking. The effects of DT exercise on cognitive function in a seated position were investigated [10,11], but few reports have examined physical function.

Notably, digital versatile discs (DVDs) or online videos are used as a means of intervention. In particular, the delivery of a DVD-containing exercise program had beneficial effects [12-14]. McAuley et al. reported that a DVD exercise intervention improved physical function in older adults [12]. Furthermore, Awick et al. found that DVD-based physical exercise enhanced self-esteem and self-efficacy [14].

To date, there have been few reports on DVD-based DT training for improving physical and cognitive function at home. Previously, we investigated the effects of DT training in a group of older adults with or without a training instructor and reported that DT programs in a seated position were effective in improving cognitive function, even without an instructor-using a DVD [11]. Furthermore, DVD exercises at home may have beneficial effects on cognitive functions [15]. However, it has been unclear whether home-based DT programs are effective for physical functions in older adults. This study aimed to investigate the effects of a DVD delivery program comprising two types of interventions on physical function: single-task (ST) and DT training. We hypothesized that exercise programs for both ST and DT training would be effective in sustaining and improving physical function and that DT training would be more effective in improving physical function than ST training because of cognitive loads.

## Materials and methods

Participants

Thirty-three participants were included in this study. Community-dwelling older adults living near the Kobe University Graduate School of Health Sciences were recruited. Recruitment was conducted by distributing information flyers around Kobe University, in local municipalities, and in public centers. The inclusion criteria for participants were as follows: (1) age between 65 and 90 years; (2) easy access to the site of this study (Kobe University) for regular interviews; (3) ability to communicate well and have a score of >26 on the Mini-Mental State Examination-Japanese (MMSE-J); and (4) no significant impairments of the extremities that affect DVD program task execution. We did not enquire whether they had participated in similar studies before participating in this study. Using G*Power 3.1 (power: 80%, effect size: large), 26 participants per group were required. However, because this study was conducted during the COVID-19 pandemic in Japan, the required number of participants could not be recruited. Therefore, this study was unavoidably conducted with a smaller number of participants. Participants who met the inclusion criteria were randomly assigned to the ST or DT training groups using block randomization.

This study conformed to the principles and guidelines described in the Declaration of Helsinki. The study protocol was approved by the Ethics Committee of the Health Sciences at Kobe University before participant recruitment (approval number: 602-3). This study was registered in the University Hospital Medical Information Network Clinical Trial Registry (UMIN000030057). The aims and contents were explained to the participants. Written informed consent was obtained if they agreed with the study and consented to participate.

Intervention

Participants assigned to either the ST or DT group were provided a DVD containing an appropriate exercise program for each group. Participants were instructed to perform exercises using DVD content at home every day for four months. They were also instructed to maintain a daily diary record to track their exercise routine. Physical assessments were conducted before and after the four-month intervention period. Their daily logs and the implementation of exercise were confirmed by visiting the university every 2 weeks. No participants missed their biweekly visits to the university for four months. It was confirmed that logs were recorded every day at that time. There were no dropouts during the four-month intervention period. Although we had planned to give simple feedback when the participants enquired regarding the exercise content throughout the intervention period, no specific questions or requests (e.g., modifications of the exercise content) were heard, and only verbal encouragement was given to a few participants.

Intervention details

In this study, ST- and DT-DVD programs, which included ST and DT training, respectively, were used as interventions (Table 1). At the beginning of the study, a DVD was given to participants. They were instructed to use the five different programs in any order to perform the exercises during the four months. The ST training was a 20-minute program comprising two parts. The first part incorporated flexibility exercises for 10 min, followed by muscle strength exercises focusing on the upper and lower extremities and trunk for 10 min. This exercise did not include any cognitive tasks and was performed as a single exercise task (e.g., compound breathing and hip flexion exercises with self-resistance). Five programs in which the second half (10 min) of the content was different were provided to avoid boredom while executing the programs.

**Table 1:** Content of ST and DT DVD training. All contents of the ST/DT DVD program consisted of approximately 20 minutes. All ST/DT DVDs include a 10-minute same-flexibility exercise program before individual ST/DT exercises. DVD, digital versatile disc

DVD program	Single task (ST) DVD	Dual task (DT) DVD
Program No. 1	A. Finger movement (grasping, opening). B. Finger coordination and pointing movement. C. Deep breathing. D. Lift the pelvis at one side.	A. Extend the little finger and extend the opposite thumb; then, extend the thumb and extend the opposite little finger, repeat. B. One fist slaps the thigh with a clenched hand, while the opposite side rubs the thigh with their fists opened. Then, swap fists and movement based on the call. C. Foot-stamping task 1. Swing their opposite upper limb, matching their stamping. 2. Swing their ipsilateral upper limb, matching their stamping. 3. Combination of aforementioned 1 and 2.
			Program No. 2	A. Swing the upper limbs forward and backwards with elbows extended. B. Shoulder rotation with hands on shoulders. C. Foot-stamping. D. Knee flexor strengthening (with resistance from their opposite limb). E. Hip abduction/adduction exercises (with resistance from their hand).	A. One fist is clenched and stretched forward, and the opposite fist is opened and placed on the chest. Then, swap fist forms and positions based on the call. B. One hand draws a line in space, and the opposite one draws a circle. C. Raising the toe while the opposite side raises the heel. Alternating and repeating that movement.
						Program No. 3	A. Press both palms on each other in front of the chest. B. Grasp each finger with the other in front of the chest and pull each hand outside. C. Standing and sitting (with upper limbs lowered along the trunk or upper limbs raised).	A. One fist slaps the thigh with a clenched fist while the opposite side rubs the thigh with their fists opened. Then, swap fist forms and movements based on the call. B. Stamping without swinging the upper limb, stamping outward, clapping hands, or interrupting the footsteps, all movements based on the call.
									Program No. 4	A. Lift and hold one leg with your hand. B. Touch the medial malleolus with the contralateral hand. C. Hip flexion exercise (with resistance by their hand). D. Knee extension. E. Ankle dorsiflexion, heel elevation. F. Ankle dorsiflexion with knee extended.	A. Only 2 fingers on 1 side are extended; all fingers on the opposite side are extended. Swap their forms based on the call. B. One lower limb forward. Simultaneously, moving their ipsilateral or opposite upper limb forward or outside based on the call.
												Program No. 5	A. Elevate and depress both shoulders simultaneously. B. Finger movement (grasping, opening). C. Finger coordination and pointing movement. D. Hip flexion exercise (with resistance from their hand). E. Knee extension. F. Ankle dorsiflexion with knee extended. G. Toe exercises (grasping, opening).	A. Raise the upper limb and lower the other (Perform based on calls). B. While foot stamping, perform the following cognitive tasks. 1. Name as many vegetables as they can. 2. Name as many prefectures as they can. 3. Name as many acquaintances as they can. 4. Add 7 to the date. Next, add 7 more to the answer. 5. Hand clapping and singing simultaneously.

The DT training was also a 20-minute program with two parts. The first part incorporated 10 minutes of flexibility exercises, identical to the ST training, and the second part comprised DT training for 10 minutes, such as a motor-cognitive task (e.g., stepping while calling an object name and hand clapping while singing) and motor tasks (e.g., moving fingers asymmetrically, asymmetrically stepping on their upper and lower limbs, and switching). Five types of DT programs were created, and the second part differed for each DVD. Both the ST and DT programs were developed by a physical therapist with more than 10 years of clinical experience, who modelled the exercises, explained the exercise details, and demonstrated each movement on both DVDs.

All the exercises were performed in a sitting position, except for *standing and sitting*, to ensure participant safety. They were designed for older adults with declining walking abilities. DT exercises while sitting effectively improved walking speed under DT conditions [16]. The program numbers that were used during the intervention period with the log were checked, and it was confirmed that the program used was not biased towards any particular program.

Outcome measures

All participants were assessed using three physical functional measurements before and after the four-month DVD exercise intervention. The outcome measurements were as follows: right and left-hand grip strength, timed-up-and-go (TUG) test, and two-step test referred to as *Locomo* in this study.

Handgrip strength is an easy and widely used assessment of global muscle strength, nutritional status, and prediction of future motor functions [17,18]. According to Mehmet et al. [18], the participants were instructed to perform grip measurements three times for both hands using digital grip measurement devices (GRIP-D, T.K.K.5401; Takei Equipment Industry Co., Niigata, Japan). The highest handgrip strength values were recorded for both hands.

The TUG test is a measurement scale generally used to assess balance and gait abilities [19,20]. In the TUG test, the participants started in a sitting position on a chair 40 cm above the ground. They were instructed to stand up, walk to a cone placed 3 m away from the chair, move around the cone, return to the chair, and sit down. These processes were initiated when a tester called out “start” as a signal. The total time required was also recorded.

The two-step test is a measurement scale used to diagnose locomotive syndrome [21]. Locomotive syndrome, a concept proposed by the Japanese Orthopedic Association, is defined as a condition involving a decline in mobility caused by dysfunction of the musculoskeletal structure and joints [22]. This dysfunction increases with age. In the two-step test, the participants were asked to take two steps with their maximum stride length. The test results were obtained by dividing the assessed measurement (cm) by the participant’s height (cm). The values were described as the *Locomo* values.

Besides these measurements, the participants were asked about their impressions of each DVD exercise program using a questionnaire after the first experience (Appendix). The ratings had three options: *difficulty*, *enjoyment*, and *tiredness*, and each DVD exercise program was noted using a five-point scale with one denoting *not at all* and five denoting *very much*. Questionnaires were administered after the first two weeks of the intervention. All assessments were conducted by assessors who were blinded to which group each participant belonged to.

Statistical analysis

Data were analyzed using SPSS version 29.0 (IBM Japan, Tokyo, Japan). Statistical analysis was performed by one of the authors other than the assessors. Since the number of subjects per group was small, nonparametric tests were used for all analyses. Regarding the difference in pre- and post-intervention between the two groups, the Mann-Whitney U test was used for continuous variables, and Fisher’s exact probability test was used for categorical variables. The Wilcoxon signed-rank test was used to compare pre- and post-intervention values for each outcome in both groups. All the analyses were two-tailed, and the significance level was set at 5%.

## Results

Characteristics of the participants

Thirty-three participants (16 males) were enrolled in this study. Seventeen participants were allocated to the ST and 16 to the DT training group, and none dropped out during this study. Furthermore, there was no missing data among all 33 participants. Hence, all data were included in the analysis. Table 2 shows the baseline characteristics of the participants. Before the intervention, there were no significant group differences in age, sex, or measurement outcomes.

**Table 2:** Characteristics of the participants at baseline. ^†^Values of the Mann-Whitney U test for continuous variables and Fisher’s exact probability test for categorical variables. A *P*-value of <0.05 was considered statistically significant. ST, single task; DT, dual task; SD, standard deviation; TUG, timed up and go; Locomo, the value of two-step value (cm) divided by the participant’s height (cm)

Variables	ST group (*n* = 17), Mean (SD)	DT group (*n *= 16), Mean (SD)	*P*^†^
Age (years)	80.18 (4.94)	77.19 (5.62)	0.116
Sex (male/female)	8/9	8/8	1.000
Right grip strength (kg)	28.29 (7.80)	28.16 (5.04)	0.787
Left grip strength (kg)	26.57 (7.43)	24.41 (4.93)	0.183
TUG test (s)	8.21 (1.38)	8.23 (1.65)	0.857
Two-step value (cm)	220.12 (32.30)	221.07 (29.14)	0.815
Locomo value	1.40 (0.18)	1.38 (0.17)	0.719

Effect of each DVD implementation

Table 3 shows the values of each measurement, including the right- and left-hand grip strength, TUG and two-step tests, and Locomo values before and after the intervention in the ST and DT groups. In the ST group, significant differences were observed in the TUG test results (*P* = 0.035), and the effect size was moderate to large (*r* = 0.51). However, no other items showed significant differences before or after the intervention. In the DT group, no significant differences were observed in any of the measurement scales before and after the intervention. After the intervention, there were no measurement scales that showed significant differences between the two groups (all *P*-values were >0.05). Adverse events, such as falls, did not occur during the intervention period.

**Table 3:** Values of the outcome measures before and after intervention and comparisons within and between groups. ^†^Values of Wilcoxon’s signed-rank test for the differences before and after the intervention. ^‡^Values of the Mann-Whitney U test for comparing the values after the intervention. A *P*-value of <0.05 was considered statistically significant. ^*^P < 0.05. ST, single task; DT, dual task; SD, standard deviation; TUG, timed up and go; Locomo, the value of two-step value (cm) divided by the participant’s height (cm)

Variables	ST group, mean (SD)			DT group, mean (SD)			*P*^‡^ (Between groups)
	Before	After	*P*^†^ (Within ST group)	Before	After	*P*^†^ (Within DT group)	
Right grip strength (kg)	28.29 (7.80)	28.79 (9.62)	0.460	28.16 (5.04)	28.21 (6.47)	0.756	0.614
Left grip strength (kg)	26.57 (7.43)	27.51 (7.81)	0.076	24.41 (4.93)	25.25 (6.66)	0.245	0.331
TUG test (s)	8.21 (1.37)	7.76 (0.92)	0.035^*^	8.23 (1.64)	8.16 (1.44)	0.642	0.471
Two-step value (cm)	220.12 (32.30)	220.04 (24.53)	0.981	221.07 (29.14)	218.99 (21.58)	0.717	0.829
Locomo value	1.40 (0.18)	1.40 (0.12)	0.906	1.38 (0.17)	1.37 (0.15)	0.796	0.540

Results of the questionnaire

The results of the questionnaire regarding the difficulty level of each DVD exercise program are presented in Table 4. Comparing the two groups, the score of *difficulty* of the DT group was significantly higher than that of the ST group (*P *= 0.004). As for the terms of *enjoyment* and *tiredness*, there were no significant differences between both groups.

**Table 4:** Results of the questionnaire regarding impressions of each DVD exercise program. ^†^Values of the Mann-Whitney U test. A *P*-value of <0.05 was considered statistically significant. ^*^P < 0.05. ST, single task; DT, dual task; DVD, digital versatile disc; SD, standard deviation

Items	ST group, mean (SD)	DT group, mean (SD)	*P*^†^
Difficulty	2.59 (0.93)	3.61 (0.67)	0.004^*^
Enjoyment	3.08 (0.40)	3.36 (0.64)	0.217
Tiredness	2.46 (1.17)	2.47 (1.10)	0.909

## Discussion

This study examined the effects of home exercise programs, provided via DVDs, on physical function and the differences in effectiveness between ST and DT. In the ST group, the time required to complete the TUG test, an indicator of walking function, decreased significantly after the intervention, whereas no effects of the intervention were observed in other measurements. While the ST group showed significant improvement in TUG, the DT group did not improve any physical measurements. Although we hypothesized that DT training would have beneficial effects on physical function, only ST training demonstrated such effects. This suggests that ST, which focuses on physical function itself, may have a more beneficial effect on physical function than DT, which requires cognitive demand, in the case of home exercise programs using DVDs. However, this result suggests that both programs may help maintain physical function, but only ST showed significant improvement in older adults in this study, even in situations or environments where frequent face-to-face or group exercises are difficult.

In the ST group, significant differences in TUG scores were observed after just 4 months of intervention, suggesting that ST may contribute to the improvement of dynamic balance and walking. Dipietro et al. noted that regular physical activity, including in cases conducted at home, can help older adults improve or delay the loss of physical function [3]. Furthermore, Shafizadeh et al. suggested that the implementation of home-based exercise programs may prevent falls [23] because of improvements in muscle strength, balance ability, and walking. In this study, the exercise component of the DVD that was provided to the ST group included factors related to overall muscle strengthening, although those exercises were conducted in a sitting position. Therefore, the possibility that this might lead to an improvement in the TUG test results due to increased muscle strength was considered. As the TUG test is a multifactorial measurement tool that includes the ability to stand up, sit down, turn around, and walk, it may more easily reflect the effect of an intervention that focuses on lower limb muscle strengthening. It has been reported that chair-based exercise improves lower limb function and balance [24,25]. This study also showed that the provision of ST-DVD programs, which are conducted in a sitting position, may have a beneficial effect on dynamic balance and walking and contribute to TUG improvement.

Previous studies have shown that exercise using DVDs enhances physical function [12,13,26]. In these studies, participants received regular phone support and advice, such as adjustments or modifications to the exercises. In the present study, the participants were asked to visit our university biweekly, have their time diaries checked, be advised on exercise methods or implementation, and be motivated to implement exercise programs. Even though this study does not require exercise modification as in previous studies, an improvement in the TUG test was noted, suggesting that DVD-based home exercises may contribute to improving or maintaining physical function even without frequent face-to-face consultations. Furthermore, none of the participants dropped out and missed their biweekly visit for the 4-month intervention period. This suggests that DVD-only programs at home, even with minimal face-to-face contact, are a feasible intervention method that can improve TUG.

The DT group showed no differences in physical function before and after intervention. Implementing DT positively affects cognitive and physical functions by simultaneously improving attention allocation and processing abilities [7-9,16,27]. In most studies, DT involved multifactorial exercise programs that combined cognitive tasks with balance, aerobic exercises, and muscle-strengthening. In this study, participants performed the DT program in a seated position to avoid the risk of falls. Because of the fewer exercise components compared with previous studies, the effect of DT shown in previous studies may not be apparent in this study. Moreover, participants of the DT group estimated the task difficulty as “more difficult” than those in the ST group. Because of the higher task demands, the DT group might have paid less attention to exercise components than the ST group in this study. Therefore, it is necessary to revise the DT program to the same level of difficulty as the ST program and to examine its effectiveness.

The variety and difficulty of DT programs are thought to be factors that make generalization difficult. Pichierri et al. suggested that cognitive-motor interventions might affect physical function; however, these effects are not clear [28]. They stated that DT programs were difficult to generalize and verify the effectiveness of those programs. From the result of the present study, it is unclear whether other types of DT programs (e.g., implemented at face-to-face conditions, etc.) would result in no significant impact on physical function.

Until approximately two years ago, social life had been drastically restricted by COVID-19, and some reports have stated that older adults are more likely to be at risk of disuse and falling in such an environment [29]. This study showed that the provision of DVD exercises may contribute to preserving and improving physical ability, even in situations where people cannot easily go out. Additionally, the fact that the average age of the participants was over 75 years, which is regarded as a very old generation in Japan, and that they maintained or improved their physical function even when targeting these older age groups, can also be considered a strength of this study. From the results of the questionnaire, participants felt that the DT programs containing cognitive tasks were more complex or difficult. However, there were no significant differences in *enjoyment *and *tiredness* between both groups, indicating that DT could be acceptable with the same level of enjoyment and tiredness as ST. Therefore, it was unlikely that the difficulty of the task affected adherence to the study, even in the participants who found the DT difficult. Finally, no adverse events such as falls occurred in this study. Further research is needed to investigate whether DVD exercise programs are effective in preventing frailty.

This study has some limitations. First, the sample size was small. During the study period, a sufficient number of participants could not be recruited because of the COVID-19 pandemic, which caused people to refrain from going outside. Second, because of the characteristics of ST and DT, it was difficult to set the same exercise load under ST and DT conditions. These results may have been influenced by the differences in the amount of exercise performed. Third, it was not clear how effective TUG improvements are for community-dwelling older adults. We should examine the clinical significance of the improvement in TUG observed in the ST-program group in the future. Fourth, the purpose of this study was to investigate the effect of the DT on physical function. It will be necessary to reconsider the content of the DT tasks and use both motor and cognitive outcomes to investigate that relationship. Fifth, the four-month intervention was conducted. So, the effect of longer exercise should be investigated. Sixth, adherence rates were checked through self-report and diary logs, which may have led to bias. Seventh, the participants who could have already participated in similar studies were included, which could lead to potential biases such as selection bias. Future studies should consider including larger samples, longer intervention periods, the employment of cognitive assessments, and standardized load matching between ST and DT.

## Conclusions

Assuming that group exercises or face-to-face instructions are difficult, such as during the COVID-19 pandemic, the effectiveness of home exercises in maintaining or improving physical function, which may help reduce frailty risk, was examined using ST or DT training in older adults. It was found that implementing the ST program, which focused on single training, such as muscle training, improved the physical function of adults aged over 75 years. The results also showed that, while DT training using a DVD did not improve physical function, it did not aggravate it. Future research should focus on optimizing the intensity and structure of DT programs, particularly to balance cognitive load with physical engagement, and should comprehensively evaluate both cognitive and physical outcomes.

## Appendices

Appendix 

**Table 5:** Questionnaire of impressions of each DVD exercise program. DVD, Digital Versatile Disc. Participants were asked to rate the three items of each DVD on a five-point scale.

Program Number	Items	Not at all		Normal		Very much
Program No.1	Difficulty	1	2	3	4	5
	Enjoyment	1	2	3	4	5
	Tiredness	1	2	3	4	5
Program No.2	Difficulty	1	2	3	4	5
	Enjoyment	1	2	3	4	5
	Tiredness	1	2	3	4	5
Program No.3	Difficulty	1	2	3	4	5
	Enjoyment	1	2	3	4	5
	Tiredness	1	2	3	4	5
Program No.4	Difficulty	1	2	3	4	5
	Enjoyment	1	2	3	4	5
	Tiredness	1	2	3	4	5
Program No,5	Difficulty	1	2	3	4	5
	Enjoyment	1	2	3	4	5
	Tiredness	1	2	3	4	5
Please describe any points if you noticed throughout all process.						
